# Strain-dependent neuronal disposition and toxicity of paclitaxel in mice

**DOI:** 10.1016/j.aspetd.2025.100005

**Published:** 2025-05-31

**Authors:** Thomas Drabison, Yue Xu, Eman A. Ahmed, Jack C. Stromatt, Nathan Colasanti, Shruthi Kandalai, Kevin M. Huang, Alex Sparreboom, Shuiying Hu, Leah M. Pyter, Eric D. Eisenmann

**Affiliations:** 1 Division of Pharmaceutics and Pharmacology, College of Pharmacy, The Ohio State University, Columbus, Ohio; 2 Comprehensive Cancer Center, The Ohio State University, Columbus, Ohio; 3 Institute for Behavioral Medicine Research, The Ohio State University, Columbus, Ohio; 4 Department of Psychiatry and Behavioral Health, The Ohio State University, Columbus, Ohio; 5 Department of Neuroscience, The Ohio State University, Columbus, Ohio

**Keywords:** DRG, Neuropathy, Paclitaxel, Pharmacokinetics, Plasma, Route, Strain

## Abstract

Paclitaxel is an antineoplastic agent that is associated with debilitating adverse effects, especially paclitaxel-induced peripheral neuropathy (PIPN). Although preclinical models have been used to characterize the mechanisms underlying PIPN, experimental differences in these models, including the dose, route, schedule of the drug, and the species and strain of the animals used, limit our ability to compare and interpret results. Furthermore, uncertainties persist regarding potential dependence of paclitaxel disposition and toxicity on the route of administration or genetic background of the animal model. In this study, we address this gap by systematically investigating the relationship among route- and strain-specific pharmacokinetic profiles, organ accumulation, and the associated toxicity phenotypes in preclinical models of PIPN. Using a panel of 7 commonly used mouse strains, we observed that the plasma pharmacokinetics of paclitaxel are strain dependent. Using 2 representative strains (C57Bl/6 and CD2F1), longitudinal studies show similar results for distribution of paclitaxel in the dorsal root ganglia (DRG) within the peripheral nervous system, with a 2-fold magnitude of difference. Ensuing studies indicated that the accumulation of paclitaxel in the DRG is correlated with susceptibility to mechanical allodynia, a key marker of PIPN. Our study implies that DRG accumulation, rather than plasma exposure, contributes to the development of PIPN, and this understanding is expected to inform the further development of translationally relevant models and effective therapeutic strategies for managing PIPN.

**Significance Statement:**

This investigation compares paclitaxel pharmacokinetics using 7 strains of mice and 2 routes of administration and longitudinally explores pharmacodynamics using 2 representative strains, which informs cross-study comparisons and the validation of translationally relevant model systems. These findings support the notion that accumulation within dorsal root ganglia, rather than plasma exposure, is correlated with the development of peripheral neuropathy. This insight is anticipated to guide the future development of effective therapeutic strategies for managing paclitaxel-induced peripheral neuropathy.

## 1. Introduction

Paclitaxel remains a widely used chemotherapeutic agent based on its efficacy in treating a broad range of solid tumors, including breast, ovarian, and lung cancer (Saloustros et al, 2008; de Juan et al, 2020; Armstrong et al, 2021; Burstein et al, 2021; Gennari et al, 2021; Remon et al, 2021; Zhu et al, 2024). Despite its clinical efficacy, paclitaxel causes adverse effects limiting its use, including paclitaxel-induced peripheral neuropathy (PIPN), a widespread, dose-limiting, and severely debilitating toxicity (Lipton et al, 1989; Marupudi et al, 2007; Carlson and Ocean, 2011; Tanabe et al, 2013; De Iuliis et al, 2015; Lu et al, 2024). PIPN is characterized by sensory disturbances, including numbness, tingling, and severe pain, presenting in a stocking-glove distribution (De Iuliis et al, 2015). This neuropathy not only impacts the quality of life for patients, but also often necessitates dose reductions or discontinuation of therapy, which may compromise the overall efficacy of cancer treatment (Pignata et al, 2006; Hershman et al, 2011).

The development of PIPN has been linked to the accumulation of paclitaxel in neuronal tissues, particularly in the dorsal root ganglia (DRG), where it causes axonal degeneration, disrupts normal neuronal function, and induces an inflammatory response (Peters et al, 2007; Scholz and Woolf, 2007; Tasnim et al, 2016; Vermeer et al, 2021). However, both the prevalence and severity of PIPN vary widely both clinically and preclinically (Smith et al, 2004; Cavaletti et al, 2024). In patients, acute PIPN occurs in about 97% of patients, with symptoms occurring within the first 24 hours of treatment (Wu et al, 2023). Acute symptoms tend to subside between treatment cycles, but the development of these acute symptoms is an indicator that a patient is more likely to develop chronic PIPN, which may persist for years or decades after treatment. About 60%–70% of patients receiving a paclitaxel-based therapy develop chronic, irreversible neuropathy (Wu et al, 2023), supporting the urgent need to develop effective strategies to predict, prevent, or treat this toxicity.

Advanced age, diabetes, obesity, hypertension, and other preexisting health conditions contribute to the progression and severity of PIPN in patients (Eckhoff et al, 2015; Sanchez-Barroso et al, 2019). Additionally, single-nucleotide polymorphisms in drug-metabolizing enzymes and transporters (DMETs) such as CYP2C8 and CYP3A4, enzymes that metabolize paclitaxel, and the paclitaxel transporters OATP1B1 and P-glycoprotein, have been associated with an increased risk of PIPN (Hertz et al, 2014; Koyanagi et al, 2019). The notion that polymorphisms in these DMETs serve as pharmacogenomic biomarkers predicting which patients are at higher risk of developing PIPN supports the thesis that DMETs are critical to the development of PIPN and that functional variation in these DMETs may impact PIPN susceptibility. We sought to explore this thesis in the context of observed variability in preclinical model systems, which have been invaluable in characterizing paclitaxel toxicities and their underlying mechanisms. However, methodological differences limit our ability to compare and interpret results reported in the published literature. In particular, it remains unclear to what extent key differences in preclinical models, including considerations of drug administration (dose, formulation, route, schedule) and animals used (sex, age, species, or strain), impact paclitaxel-induced toxicities. In this study, we systematically investigated the influence of mouse strain on the pharmacokinetics and pharmacodynamics of paclitaxel, using a panel of commonly used models.

## 2. Materials and methods

### 2.1. Preparation and dilution of paclitaxel

Paclitaxel was purchased as the commercially available pharmaceutical formulation Taxol, which is available as a 6-mg/mL solution that uses 50% Kolliphor EL (polyoxyethylene glycerol triricinoleate 35; formerly known as Cremophor EL) and 50% ethanol as solvents, or powder (LC-Labs). For administration, Taxol was diluted 1:3 in 0.9% NaCl saline and subsequently mixed to form a 2 mg/mL dosing solution. For paclitaxel compounded in-house, which was necessary to normalize the amount of Kolliphor EL when mice were administered different doses of paclitaxel, the powder was weighed out and dissolved in equal parts Kolliphor EL and anhydrous ethanol at 3 or 6 mg/mL. This was then diluted 1:3 in 0.9% NaCl to form a 1- or 2-mg/mL dosing solution. All mice were dosed with 5 *μ*L/mg of body weight. The control vehicle for this study was 1:1 Kolliphor EL:ethanol, diluted 1:3 in normal saline.

### 2.2. Animals

For all experiments, 8–16-week-old, female, age-matched mice were used. Animals were housed in standard temperature-controlled cages with access to a standard diet and water *ad libitum* in a 12 h light cycle. Housing conditions and handling were approved by the University Laboratory Animal Resources Animal Care and Use Committee at The Ohio State University (OSU), under approved animal protocols (2015A00000101-R2 and 2024A00000067). Wild-type S129, C57Bl/6, and FVB mice were purchased from Taconic Biosciences (Taconic Biosciences, IN), BALBc and CD2F1 mice from Charles River, DBA/1J mice from Jackson Laboratory (Bar Harbor, ME), and NSG (NOD.Cg-Prkdcscid Il2rgtm1Wjl/SzJ) mice, originally from Jackson Laboratory, from the Preclinical Therapeutics Mouse Modeling Shared Resource at OSU.

### 2.3. Pharmacokinetic studies

Pharmacokinetic studies were performed following an established protocol (Leblanc et al, 2018a). Paclitaxel was administered via a single intravenous or intraperitoneal injection at doses of 5 or 10 mg/kg. To capture the peak concentration (C_max_) and terminal phase with each route, samples were collected at 3, 7, 15, 30, 60, and 120 minutes (intravenous) and 15, 30, 60, 120, and 240 minutes (intraperitoneal) based on historical data describing the pharmacokinetics with each route (Gelderblom et al, 2002). Whole blood samples (20–30 *μ*L) were collected from the submandibular vein for the initial 3 time points, from the retro-orbital sinus vein (20–30 *μ*L) for the subsequent 2 time points, and cardiac puncture (100–1000 *μ*L) at the terminal time point. Blood samples were centrifuged at 13,000 × *g* for 5 minutes, and the plasma supernatants were collected and stored at −80 °C until analysis. DRG were collected at the endpoint, and, to prevent continuing metabolic activity, tissues were snap-frozen using dry ice. DRG tissues were homogenized in 20 *μ*L of LC-MS-grade water with 5-mm stainless steel beads (Qiagen) and processed for 4 minutes at 40 Hz. Finally, the beads were removed, and the homogenized samples were centrifuged at 13,000 × *g* for 5 minutes, and the supernatants were collected and stored at −80 °C until analysis.

### 2.4. Paclitaxel measurement

Paclitaxel concentrations in plasma and DRG were measured using a validated liquid chromatography-tandem mass spectrometry (LC-MS/MS) method, which was adapted from previously published protocols (Mortier et al, 2004; Leblanc et al, 2018b). Briefly, a Vanquish Ultra-High-Performance Liquid Chromatography system, coupled with a TSQ Quantiva triple quadrupole mass spectrometer (Thermo Fisher Scientific), was used to quantify paclitaxel (purity 99.99%, Selleckchem) and its internal standard, [2H5]-Paclitaxel (purity > 95%, Alsachim). Chromatographic separation was performed on an Accucore aQ column (150 mm × 2.1 mm, 2.6 *μ*m) fitted with a C18 Aquasil guard cartridge (Thermo Fisher Scientific), with the column maintained at 20 °C. The mobile phase consisted of 0.1% formic acid in LC-MS grade water (solvent A) and a 50:50 mixture of 0.1% formic acid in acetonitrile and methanol (solvent B), with a total run time of 4.6 minutes.

Mass spectrometry conditions were optimized in positive ionization mode with sodium adduct (Mortier et al, 2004) to achieve stable and maximum responses for paclitaxel. Precursor and product ions were monitored to detect and quantify paclitaxel (876.3→ 308.06) using [^2^H_5_]-Paclitaxel (881.4/313.07) as the internal standard. A 9-point calibration curve, ranging from 1.25 to 1000 ng/mL, was generated. Over a period of 4 days, 2 calibration curves were constructed daily, demonstrating satisfactory linearity with a coefficient of determination (r^2^) exceeding 0.99. Quality control samples were prepared at 5 concentrations. The accuracy (bias, %) for lower limit of quantification samples was determined to be 5.9%. The 4 quality control levels (lower quality control, middle quality control, high quality control, and above upper limit quality control) exhibited an overall accuracy (bias, %) ranging from −6.7% to 9.1%. Data acquisition and processing were conducted using Thermo Scientific Xcalibur software (version 4.4.16.14). The method was developed and validated in compliance with Food and Drug Administration (FDA) bioanalytical method validation guidelines (https://www.fda.gov/regulatory-information/search-fda-guidance-documents/bioanalytical-method-validation-guidance-industry).

For sample preparation, paclitaxel was extracted from mouse plasma and DRG supernatants using protein precipitation. A 10-*μ*L sample was mixed with 5 *μ*L of internal standard solution (10 *μ*g/mL) and 85 *μ*L of methanol. The mixture was vortexed, followed by centrifugation at 13,000 rpm for 10 minutes at 4 °C. A 70-*μ*L aliquot of the supernatant was transferred to a 96-well plate, sealed, and 5 *μ*L was analyzed.

### 2.5. Pharmacokinetic data analysis

Noncompartmental analysis was performed to determine the pharmacokinetic parameters for paclitaxel using Lixoft PKanalyix (Antony). Values for C_max_ were determined by visual inspection of the data from the log concentration versus time curves. The linear trapezoidal rule was used to obtain the area under the plasma concentration-time curve (AUC) from time zero to the time of the last measurable concentration (AUC_0–last_) or extrapolated to infinity (AUC_inf_).

### 2.6. Peripheral neuropathy assessment

Mice received a single dose of paclitaxel (5 or 10 mg/kg; intravenous) or vehicle. Nocifensive behavior was assessed following mechanical stimulation in a von Frey hairs test (VFH) to assess mechanical allodynia at 2, 6, 24, 48, and 72 hours after drug administration. Mice were housed individually in elevated cages with unobstructed access to their hind paws and acclimated to the VFH testing apparatus 1 day before the experiment. Baseline measurements were obtained before dosing. Behavioral testing was conducted in a quiet room following procedures previously described (Chiorazzi et al, 2018; Huang et al, 2020).

Mice were placed in elevated enclosures and allowed to acclimate for 1 hour before initiation of testing. A filament connected to an electronic Anesthesiometer (MouseMet Topcat Metrology) was applied perpendicularly to the plantar surface of the hind paw with a constant force. Upon spontaneous hind paw withdrawal, the stimulus was stopped and the gram force of the pressure to withdrawal was recorded. This procedure was assessed 3 times by applying to the midplantar region of each hind paw from beneath the mesh floor and with intervening intervals of a few seconds. Values for each animal were then used to average across groups to yield a mean group threshold response, an upper cutoff limit of 6 g was set to prevent tissue damage. Group compositions in the VFH tests were blinded to the analyst.

### 2.7. Data analysis

All data represent the mean ± SD before and/or after normalization to baseline values and are expressed as a percentage unless stated otherwise. An unpaired 2-sided Student’s *t* test with Welch’s correction was used for normally distributed data, and a Kolmogorov-Smirnov test was used for nonnormally distributed data for comparisons between 2 groups. An ordinary one-way ANOVA was used for comparing more than 2 groups. Behavioral data were analyzed using a two-way and three-way ANOVA with Bonferroni’s post hoc test, repeated across time points and groups. A *P* value of <.05 was used as the statistical cutoff across all analyses.

## 3. Results

### 3.1. Paclitaxel pharmacokinetics depend on mouse strain

**Fig. 1.**
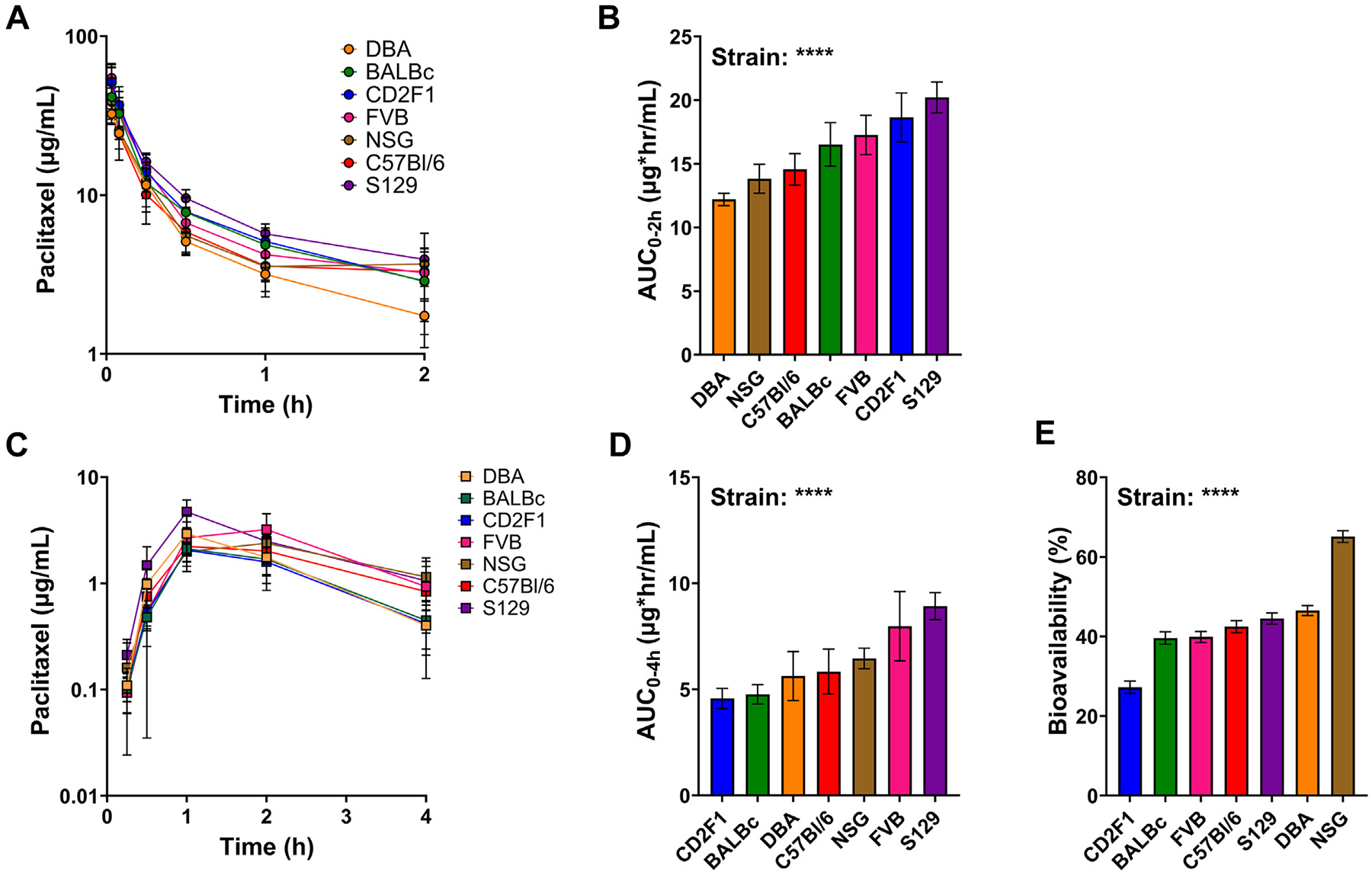
Pharmacokinetics of paclitaxel across different strains of mice. Plasma concentration-time profiles of paclitaxel at 10 mg/kg (A) administered intravenously or (C) intraperitoneally. AUC_last_ was calculated at (B) 2 hours (intravenous) or (D) 4 hours (intraperitoneal) using noncompartmental analysis (E) Bioavailability calculated as a ratio of AUC_inf_ for both intravenous and intraperitoneal paclitaxel. (n = 5 per group, error bars represent SD) ****P < .0001

To investigate the hypothesis that the pharmacokinetic profile of paclitaxel is strain-dependent, we analyzed drug disposition properties in 7 commonly used and commercially available mouse strains. Based on data suggesting that this dose causes PIPN (Leblanc et al, 2018b), we administered a single 10-mg/kg dose of paclitaxel intravenously (Fig. 1A) or intraperitoneally (Fig. 1C), collected blood samples, and analyzed plasma concentrations of paclitaxel (Table 1). Consistent with the thesis that mouse strain might influence distribution and elimination (Barr et al, 2020), we observed statistically significant strain-dependent differences in the pharmacokinetic profile of paclitaxel after intravenous and intraperitoneal administration. When the impact of strain on C_max_ and AUC was compared using a one-way ANOVA, there were significant main effects for both intravenous (*P* < .001 C_max_; *P* < .001 AUC_0–2h_) and intraperitoneal administration (*P* < .001 C_max_; *P* < .001 AUC_0–4h_) (Table 1). Following intravenous administration, there was a 1.7-fold difference between the lowest (32.4 ± 4.63 *μ*g/mL; DBA) and highest C_max_ (54.4 ± 11.69 *μ*g/mL; FVB) and a 1.6-fold difference between the lowest (12.2 ± 1.10 *μ*g*h/mL; DBA) and highest AUC_0–2h_ (20.2 ± 2.71 *μ*g*h/mL; S129) (Fig. 1B and Table 1). For intraperitoneal administration, there was a 2.3-fold difference between the lowest (2.1 ± 0.18 *μ*g/mL; CD2F1) and highest C_max_ (4.7 ± 1.36 *μ*g/mL; S129) and a 2.0-fold difference between the lowest (4.6 ± 1.07 *μ*g*h/mL; CD2F1) and highest AUC_0–4h_ (8.9 ± 1.43 *μ*g*h/mL; S129) (Fig. 1D). In a two-way ANOVA of C_max_ and AUC_inf_ values (route × strain), for intraperitoneal and intravenous, there was a significant main effect of strain (*P* < .0001, *P* < .0001, respectively) and route (*P* < .0001, *P* < .0001, respectively). Based on the calculated AUC_inf_ values, we compared intravenous and intraperitoneal exposure and calculated the intraperitoneal bioavailability of paclitaxel, which was also strain-dependent. When the impact of strain on bioavailability was compared using a one-way ANOVA, there was a significant main effect (*P* < .0001) (Fig. 1E). Bioavailability ranged from 27% in CD2F1 mice to 65% in NSG mice.

### 3.2. Strain-dependent accumulation of paclitaxel within the DRG

**Fig. 2.**
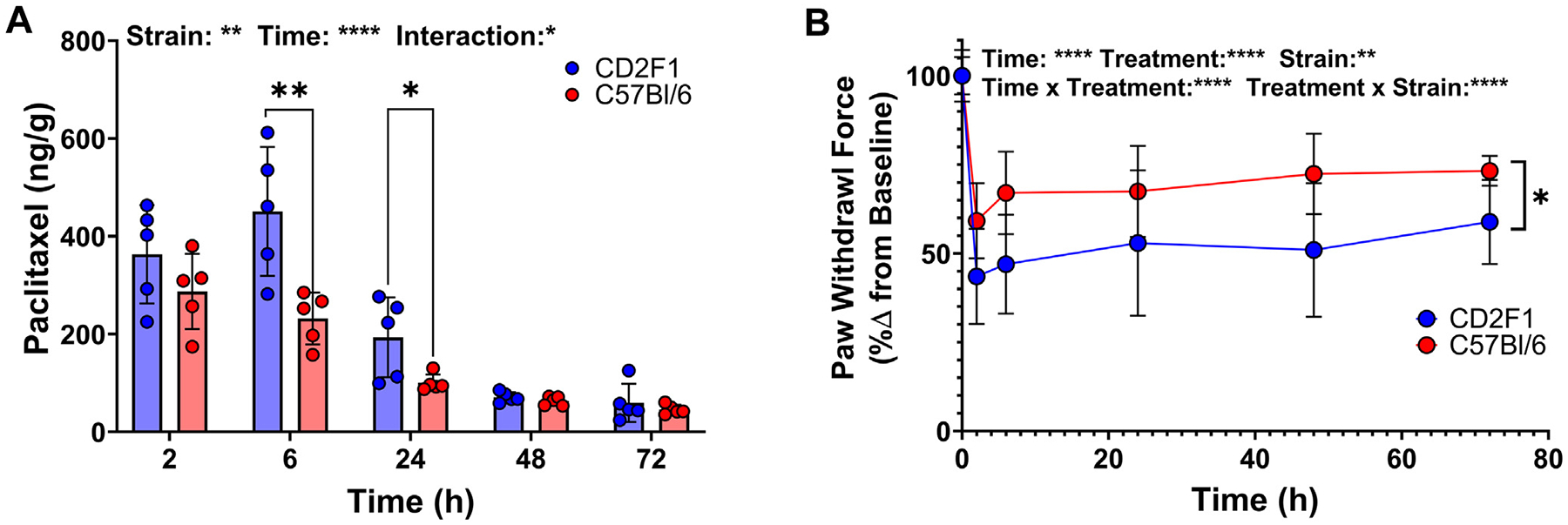
Differential paclitaxel accumulation in DRG tissues and toxicity endpoints. (A) Paclitaxel concentrations in DRG from CD2F1 and C57Bl/6 mice following a single intravenous bolus of paclitaxel at a dose of 10 mg/kg as determined by LC-MS/MS and normalized to tissue weight (n = 5 per group, error bars represent SD). (B) VFH in CD2F1 and C57Bl/6 mice following a single intravenous bolus of paclitaxel at a dose of 10 mg/kg, expressed as a percent change from baseline (n = 6 per group, error bars represent SD). *P < .05, **P < .01, ***P < .001, ****P<.0001.

Based on reported differences in susceptibility to PIPN between strains of mice (Smith et al, 2004), our observed strain-dependent differences in plasma pharmacokinetics of paclitaxel, and the notion that systemic concentrations drive organ accumulation (Sparreboom et al, 1996b), we hypothesized that paclitaxel exposure within the DRG, believed to be the primary site of injury for PIPN (Xu et al, 2022), is also strain-dependent. To test this hypothesis, we first selected 2 ‘high AUC’ strains (CD2F1 and S129) and 2 ‘low AUC’ strains (DBA and C57Bl/6) (Fig. 1B) and conducted an initial screen by assessing paclitaxel concentrations within the DRG 24 h after a single 10-mg/kg intravenous bolus. A one-way ANOVA revealed a significant main effect of mouse strain (*P* = .0031), with the greatest difference observed between CD2F1 and C57Bl/6 mice (2.1-fold; *P* = .0028) (Supplemental Fig. 1). Surprisingly, differences in DRG accumulation were larger than differences in plasma exposure based on fold change, suggesting a disconnect between plasma pharmacokinetics and DRG accumulation. Based on DRG accumulation of paclitaxel, we selected CD2F1 (high DRG accumulation) and C57BL/6 (low DRG accumulation) as representative strains to further characterize our hypothesis that DRG accumulation of paclitaxel is strain-dependent. A 10-mg/kg intravenous bolus was administered to CD2F1 and C57Bl/6, and concentrations of paclitaxel in DRG were analyzed from 2 to 72 hours (Fig. 2A). Consistent with our hypothesis, a two-way ANOVA (strain × time) revealed significant main effects of strain (*P* = .0017) and time (*P* < .0001) and a significant interaction between these factors (*P* = .0105). The largest differences in DRG exposure were observed at 6 hours (*P* = .0087) and 24 hours (*P* = .038) (Fig. 2A).

Based on observed differences in DRG accumulation, we tested the hypothesis that CD2F1 and C57Bl/6 mice are differentially sensitive to the development of PIPN. We performed an experiment assessing mechanical allodynia at parallel time points after administering a single 10-mg/kg intravenous bolus of paclitaxel or vehicle to CD2F1 and C57Bl/6 mice (Fig. 2B and Supplemental Fig. 2). Regardless of strain, the control vehicle did not independently contribute to changes in paw sensitivity in mice (Supplemental Fig. 2). Consistent with the notion that higher paclitaxel levels in the DRG would exacerbate PIPN, we found that CD2F1 mice treated with paclitaxel had increased paw sensitivity from baseline compared to C57Bl/6 mice; a three-way ANOVA (treatment × strain × time) found a significant treatment × strain interaction (*P* = .0039). Consistent with this analysis, quantifying toxicity using the area over the curve (AOC) for mechanical allodynia (Mathijssen et al, 2002), CD2F1 mice had an AOC of 3411 ± 1119 g*h, whereas C57Bl/6 mice showed an AOC of 2152 ± 380 g*h (unpaired *t* test; *P* = .04). This correlation between elevated paclitaxel accumulation in the DRG and increased peripheral neuropathy severity suggests that susceptibility to PIPN may be influenced by intrinsic strain-specific pharmacokinetic differences.

### 3.3. Paclitaxel accumulation in the DRG as a potential driver of PIPN susceptibility

To further assess whether differences in susceptibility to PIPN are driven by strain-dependent differences in paclitaxel accumulation, we adjusted the dosing regimen to normalize DRG paclitaxel concentrations between C57Bl/6 and CD2F1 mice and evaluated PIPN in the same cohorts. Given the dose-linear pharmacokinetics of paclitaxel in murine tissues between 2 and 20 mg/kg (Sparreboom et al, 1996b) and the observation that DRG levels were twice as high in CD2F1 mice given the same dose as C57Bl/6 mice (Fig. 2A), C57Bl/6 mice were administered a dose of 10 mg/kg and CD2F1 mice received 5 mg/kg (Sparreboom et al, 1996b). To control for the potential confounding effect of Kolliphor EL, a component of the vehicle used for paclitaxel that could independently contribute to peripheral neuropathy (Mielke et al, 2006) and causes paclitaxel to exhibit nonlinear plasma pharmacokinetics (Sparreboom et al, 1996a), dosing solutions were standardized so that each mouse received equivalent volumes of Kolliphor EL relative to body weight (0.84 mg Kolliphor EL/g body weight). To determine whether the plasma pharmacokinetics of paclitaxel are different between the diluted commercial formulation (ie, from Taxol) or an in-house compounded formulation (ie, from powder), we conducted an experiment comparing the 2 formulations. As expected, the pharmacokinetics of paclitaxel were similar between these 2 formulations (Supplemental Fig. 3 and Supplemental Table 1), which supported our experiment normalizing the amount of Kolliphor EL by dissolving paclitaxel powder. In our dose modulation study, the plasma pharmacokinetics of paclitaxel were significantly different between CD2F1 mice administered 5 mg/kg and C57Bl/6 mice administered 10 mg/kg (Supplemental Fig 4. and Supplemental Table 2), with *t* tests revealing significant differences observed in both AUC_0–2h_ (*P* < .0001) and C_max_ (*P* < .0001). Our data are generally consistent with the notion that proportionally larger amounts of Kolliphor EL increase paclitaxel plasma exposure and that the presence of Kolliphor EL causes nonlinear increases in paclitaxel plasma exposure with increasing doses (Sparreboom et al, 1996a). Nonetheless, organ accumulation of paclitaxel in the presence of Kolliphor EL is generally proportionally increasing with an increase in dose (Sparreboom et al, 1996b), which is supported by our data demonstrating that reducing the dose administered to CD2F1 mice by half resulted in a 50% decrease in DRG accumulation.

**Fig. 3.**
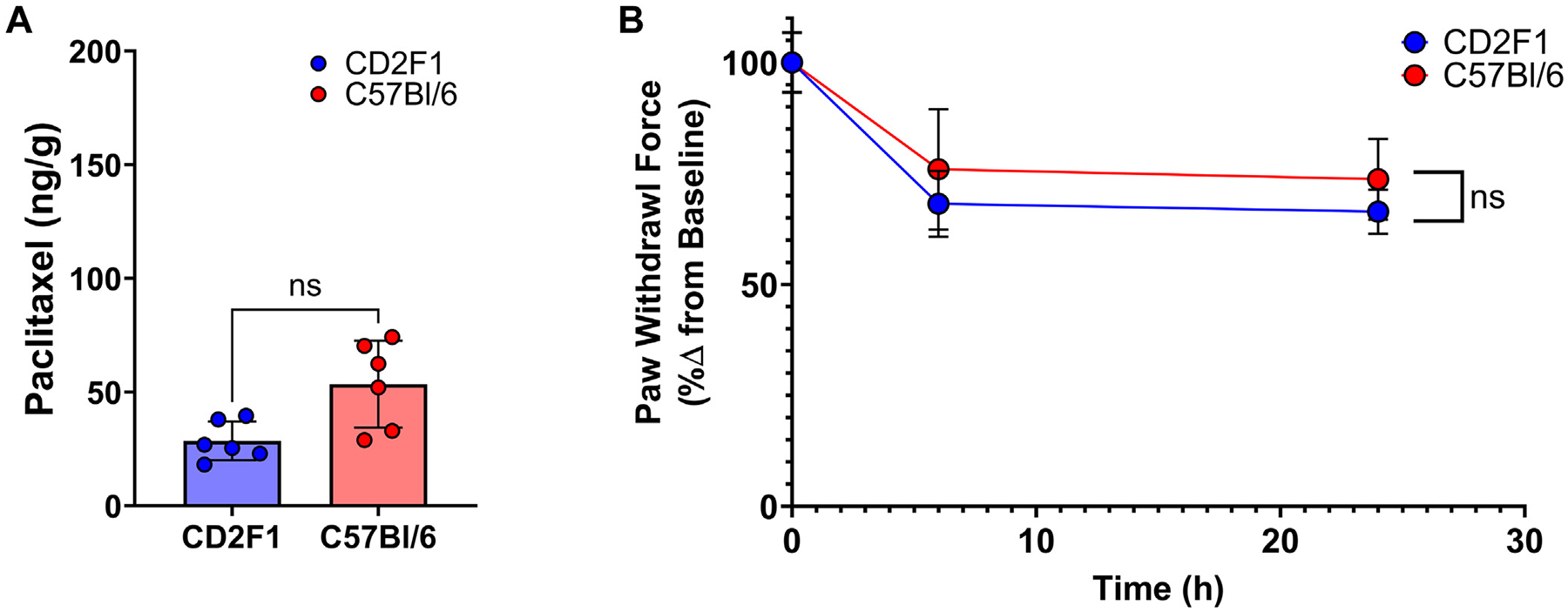
Dose modulation to achieve comparable DRG exposure to paclitaxel. (A) Paclitaxel concentrations in DRG from CD2F1 and C57Bl/6 mice at 24 h following a single intravenous bolus of paclitaxel at a dose of 5 mg/kg (CD2F1) and 10 mg/kg (C57Bl/6) as determined by LC-MS/MS and normalized to tissue weight (*n* = 6 per group, error bars represent SD). (B) VFH in the same cohort of CD2F1 and C57Bl/6 mice at baseline and 6 and 24 hours following a single intravenous bolus of paclitaxel at a dose of 5 mg/kg (CD2F1) and 10 mg/kg (C57Bl/6), expressed as a percent change from baseline (*n* = 6 per group, error bars represent SD).

Based on these preliminary studies, we administered paclitaxel intravenously to CD2F1 mice (5 mg/kg) and C57Bl/6 mice (10 mg/kg), and we found comparable DRG paclitaxel concentrations at the 24-hour time point (*P* > .05) (Fig. 3A). Behavioral assessment of pain response in this same cohort revealed no significant difference in paw sensitivity between C57Bl/6 and CD2F1 mice at both 6- and 24-hour time points (Fig. 3B), in contrast to the previously observed differences when equal doses were administered. These findings demonstrate that the strain-specific susceptibility to PIPN is correlated with strain-dependent differences in paclitaxel accumulation within the DRG, suggesting that inherent biological variations between mouse strains impact paclitaxel pharmacokinetics, organ accumulation, and toxicity.

## 4. Discussion

Based on the observed differences in susceptibility to PIPN within and between preclinical studies that use different strains of mice (Smith et al, 2004; Cavaletti et al, 2024) that cannot be fully explained by baseline behavioral differences between inbred mouse strains in measures of nociception, including mechanical hypersensitivity (Mogil et al, 1999a,b; Lariviere et al, 2002), we hypothesized that strain-dependent pharmacokinetics may cause differences in susceptibility to PIPN. In this study, we demonstrate that paclitaxel plasma pharmacokinetics, organ accumulation, and neuropathy depend on the strain of mouse. This is the first report that comprehensively analyzes pharmacokinetic differences between strains of mice and the potential impact of these differences on paclitaxel toxicity. Our results suggest that mouse strain is an important methodological consideration in the development and interpretation of preclinical models of PIPN. These previously unexplored differences have limited the ability to accurately compare investigations using different strains of mice.

In the current study, we selected a panel of 7 strains based on their relevance to the study of paclitaxel pharmacokinetics, including existing preclinical models, transgenic strains, and historical data. C57Bl/6, BALBc, and S129 mice have been used extensively to develop preclinical models of paclitaxel-related toxicities (Loman et al, 2019, 2024; Kawashiri et al, 2021). NSG mice are transgenic mice that carry 2 mutations on the NOD/ShiLtJ background that make them severely immunodeficient, which has made them invaluable to the development of xenograft models to study anticancer activity (Ito et al, 2023). FVB and DBA mice are the background strain for mice deficient for genes encoding DMETs involved in paclitaxel pharmacokinetics, including CYP3A (van Herwaarden et al, 2007) and OATP1B2 (Zaher et al, 2008), respectively. The CD2F1 strain was previously used to determine the intraperitoneal bioavailability of paclitaxel (Eiseman et al, 1994).

We used this panel of 7 strains to characterize the pharmacokinetics of paclitaxel after intravenous or intraperitoneal administration. The AUC_0–2h_ of intravenous 10 mg/kg paclitaxel ranged between 12.2 – 20.2 *μ*g*h/mL, which is consistent with the established pharmacokinetic profile of intravenous paclitaxel reported previously (Eiseman et al, 1994; Sparreboom et al, 1996a,^1998^; Nieuweboer et al, 2014). The AUC_0–4h_ of intraperitoneal 10 mg/kg paclitaxel ranged between 4.5–8.9 *μ*g*h/mL and the intraperitoneal bioavailability of paclitaxel ranged from 27% to 65%. Although paclitaxel is rarely given intraperitoneally in patients (Sugarbaker, 2021), this administration route is widespread in preclinical models of paclitaxel toxicity (Smith et al, 2004; Toma et al, 2017; Loman et al, 2019). The intraperitoneal bioavailability of paclitaxel has not previously been comprehensively evaluated, despite discrepancies in the literature suggesting intraperitoneal bioavailability may be as low as 10% in one strain (Eiseman et al, 1994) or closer to unity in another (Innocenti et al, 1995). Our data confirm this variability and suggest that strain-dependent intraperitoneal bioavailability could impact organ accumulation or sensitivity to paclitaxel-induced toxicity. Previous investigations have used intraperitoneal administration of paclitaxel to assess strain-dependent differences in PIPN severity (Smith et al, 2004). However, the absence of pharmacokinetic data precludes direct comparisons between pharmacokinetic data and behavioral phenotypes. To generate proof-of-concept, we have focused on these considerations in the context of intravenous paclitaxel. The observed 2-fold variability in plasma exposure between strains is both significant and consistent with the relatively scarce literature describing strain-dependent pharmacokinetic differences (Löfgren et al, 2004; McCarthy et al, 2004; Guo et al, 2006; Barr et al, 2020). Despite observing plasma pharmacokinetic variability within a 2-fold range, a cutoff previously used (Daublain et al, 2017; Barr et al, 2020) based on FDA guidance for significant hepatic impairment effects (https://www.fda.gov/regulatory-information/search-fda-guidance-documents/pharmacokinetics-patients-impaired-hepatic-function-study-design-data-analysis-and-impact-dosing-and), we now document that there are also strain-dependent differences in organ accumulation and toxicity. This suggests that caution should be exercised with respect to mouse strain selection when developing or interpreting data generated using preclinical models (Barr et al, 2020). Our data imply that there are strain-dependent differences in DMETs governing paclitaxel disposition, and characterizing these differences is the subject of our ongoing research. Nonetheless, we have applied the observed strain-dependent differences to determine the relative importance of plasma exposure and organ accumulation in driving PIPN.

Our data suggest that higher DRG exposure, rather than plasma exposure, increases paclitaxel neurotoxicity and that plasma pharmacokinetics are not necessarily a direct predictor of whether paclitaxel will precipitate neuropathy. Based on the notion that paclitaxel dose density (eg, cumulative exposure, higher doses, and additional treatment cycles) is associated with a higher incidence of PIPN (Postma et al, 1995; Briasoulis et al, 2002; Mielke et al, 2003, 2006; Klein and Lehmann, 2021), paclitaxel plasma exposure has previously been explored as a potential predictive marker of PIPN (de Graan et al, 2013). Although some studies have correlated PIPN with certain plasma pharmacokinetic parameters of paclitaxel in patients, including threshold concentrations (Mielke et al, 2005) and C_max_ (Hertz et al, 2018), these correlations have been disputed (Papadopoulos et al, 2001) and remain controversial (Sun et al, 2024). To shed light on the reported discrepancies, we used different doses of paclitaxel to titrate strain-dependent toxicity, and found that DRG exposure, rather than plasma exposure, was predictive of PIPN. This thesis is consistent with previous observations that inhibition of paclitaxel uptake into DRG is associated with amelioration of PIPN without simultaneously impacting measures of systemic exposure in plasma (Leblanc et al, 2018b; Klein et al, 2023). The absence of a direct correlation between plasma concentrations of paclitaxel and accumulation at the site of injury within the peripheral nervous system implies that measures of systemic exposure from a single dose are unlikely to serve as suitable predictive biomarkers of PIPN. Given that direct measurement of paclitaxel levels in the DRG is not clinically feasible, future research seeking to accurately predict PIPN and improve patient outcomes should prioritize the discovery and validation of other informative biomarkers [eg, neurofilament light chain (Andersen et al, 2024) or a-tocopherol (Li et al, 2023)]. Although decreasing the dose of paclitaxel received by a patient could decrease the development or severity of PIPN, which will be reflected in paclitaxel plasma exposure, this is likely an indirect measure of accumulation at the site of injury, rather than a leading metric. This disconnect could, in part, explain why therapeutic drug monitoring algorithms for paclitaxel need to incorporate additional patient-specific clinical factors, including assessment of neutropenia, rather than targeting a specific window of plasma concentrations (Hertz et al, 2024). Targeting a specific plasma concentration is further complicated by the nonlinear plasma pharmacokinetics of paclitaxel caused by the excipient Kolliphor EL (Sparreboom et al, 1996a), depending on the dose, infusion duration, and schedule employed (van Zuylen et al, 2001).

Kolliphor EL is used as a solvent in the conventional formulation of paclitaxel [Taxol; (Adams et al, 1993)], and this excipient causes various side effects, including hypersensitivity reactions, and interacts with paclitaxel such that paclitaxel exhibits nonlinear plasma pharmacokinetics (Gelderblom et al, 2001). Based on these considerations, alternative paclitaxel formulations have been developed (Terwogt et al, 1997), including an FDA-approved albumin nanoparticle-based formulation (nab-paclitaxel; Abraxane) (Singla et al, 2002). This solvent-free formulation overcomes several of the limitations associated with Kolliphor EL and allows for higher doses to be administered more quickly with no premedication for hypersensitivity reactions (Yamamoto et al, 2011). Despite early evidence that nab-paclitaxel has improved efficacy and reduced toxicity compared with Taxol (Gradishar et al, 2005), current evidence suggests that, despite improvements in efficacy, PIPN may actually be worse with nab-paclitaxel (Guo et al, 2019; Kida et al, 2024). Consistent with these data and the present investigation, the extent to which paclitaxel accumulates in DRG is increased with nab-paclitaxel relative to Taxol despite decreases in plasma exposure with nab-paclitaxel (Girdenyte et al, 2024), which is possibly due to the inhibition of relevant DRG uptake mechanisms by Kolliphor EL (Nieuweboer et al, 2014). The present findings support the notion that increased DRG accumulation with nab-paclitaxel is responsible for observed increases in neuropathy and that decreasing this accumulation could be an effective preventive strategy. We are actively pursuing this hypothesis by comparing DRG accumulation and PIPN associated with Taxol and Abraxane as a function of dose, treatment frequency, and route of administration (Ouyang et al, 2023; Cao et al, 2024; Cavaletti et al, 2024; Lu et al, 2024) to rationally inform the development of strategies to prevent or treat PIPN.

The findings reported here serve as an important foundation for better understanding the utility of preclinical models as translationally suitable tools to evaluate the plasma pharmacokinetics, tissue distribution and toxicity associated with paclitaxel. In particular, our studies imply that genetic differences between mouse strains impacting DRG accumulation influence the development of PIPN. Future work will take advantage of this knowledge to inform the development of therapeutic strategies seeking to mitigate PIPN and improve patient outcomes.

## Supplementary Material

1

This article has supplemental material available at aspetdiscovery.org.

## Figures and Tables

**Table 1:** Paclitaxel pharmacokinetic parameters

Mouse Strain	Sex	*n*	Route	C_max_ ± SD (*μ*g/mL)	AUC_last_ ± SD (*μ*g × h/mL)	AUC_inf_	F^a^
S129	F	5	IP	4.7 ± 1.36	8.9 ± 1.43	12.1	
	F	5	IV	53.8 ± 10.5	20.2 ± 2.71	27.2	.45
BALBc	F	5	IP	2.1 ± 0.22	4.8 ± 1.03	8.3	
	F	5	IV	41.5 ± 13.01	16.5 ± 3.82	21	.40
C57B1/6	F	5	IP	2.2 ± 0.94	5.8 ± 2.37	8.7	
	F	5	IV	51.2 ± 16.19	14.6 ± 2.75	20.4	.42
CD2F1	F	5	IP	2.1 ± 0.18	4.6 ± 1.07	7	
	F	5	IV	51.7 ± 11.76	18.6 ± 4.32	25.8	.27
DBA	F	5	IP	2.9 ± 1.48	5.6 ± 2.57	6.8	
	F	5	IV	32.4 ± 4.63	12.2 ±1.10	14.7	.46
FVB	F	5	IP	3.2 ± 1.45	8.0 ± 3.64	9.7	
	F	5	IV	54.5 ± 11.69	17.3 ±3.47	24.4	.40
NSG	F	5	IP	2.4 ± 0.69	6.5 ± 1.07	11.1	
	F	5	IV	38.6 ± 8.56	13.8 ±2.53	17	.65

AUC_0–last_, area under the concentration-time curve (AUC) from time zero to the last observed time point (2 hours for IV, 4 hours for IP); AUC_inf_, AUC from time zero extrapolated to infinity; C_max_, maximum plasma concentration.

a Bioavailability (F) calculated using AUC_inf_.

## Data Availability

The authors declare that all the data supporting the findings of this study are available within the paper and its Supplemental Data.

## Abbreviations

AOC: area over the curve
AUC: area under the curve
AUC_0–last_: area under the curve from time zero to the time of the last measurable concentration
AUC_inf_: area under the curve extrapolated to infinity
C_max_: peak plasma concentration
CYP: cytochrome P450
DMET: drug-metabolizing enzymes and transporters
DRG: dorsal root ganglion
FDA: United States Food and Drug Administration
LC: liquid chromatography
MS: mass spectrometry
OATP: organic anion transporting polypeptide
PIPN: paclitaxel-induced peripheral neuropathy
VFH: von Frey hairs test
