# Supplementary material for: Strain-dependent neuronal disposition and toxicity of paclitaxel in mice
Source: ASPET Discov. Author manuscript; Available in PMC 2026 Jun 24. (PMC13290288; doi:10.1016/j.aspetd.2025.100005)
Supplement: 1 [file NIHMS2176670-supplement-1.docx]

**Supplemental Information – ASPET Discovery**

**Strain-Dependent Neuronal Disposition and Toxicity of Paclitaxel in Mice**

Thomas Drabison^1^, Yue Xu^1^, Eman A. Ahmed^1^, Jack C. Stromatt^1^, Nathan Colasanti^1^, Shruthi Kandalai^1^, Kevin M. Huang^1^, Alex Sparreboom^1,2^, Shuiying Hu^1,2^, Leah M. Pyter^3,4,5^, and Eric D. Eisenmann^1,2,#^

**Authors’ Affiliations:**

^1^Division of Pharmaceutics and Pharmacology, College of Pharmacy, The Ohio State University, Columbus, OH, USA

^2^Comprehensive Cancer Center, The Ohio State University, Columbus, OH, USA

^3^Institute for Behavioral Medicine Research, The Ohio State University, Columbus, OH, USA

^4^Department of Psychiatry and Behavioral Health, The Ohio State University, Columbus, OH, USA

^5^Department of Neuroscience, The Ohio State University, Columbus, OH, USA


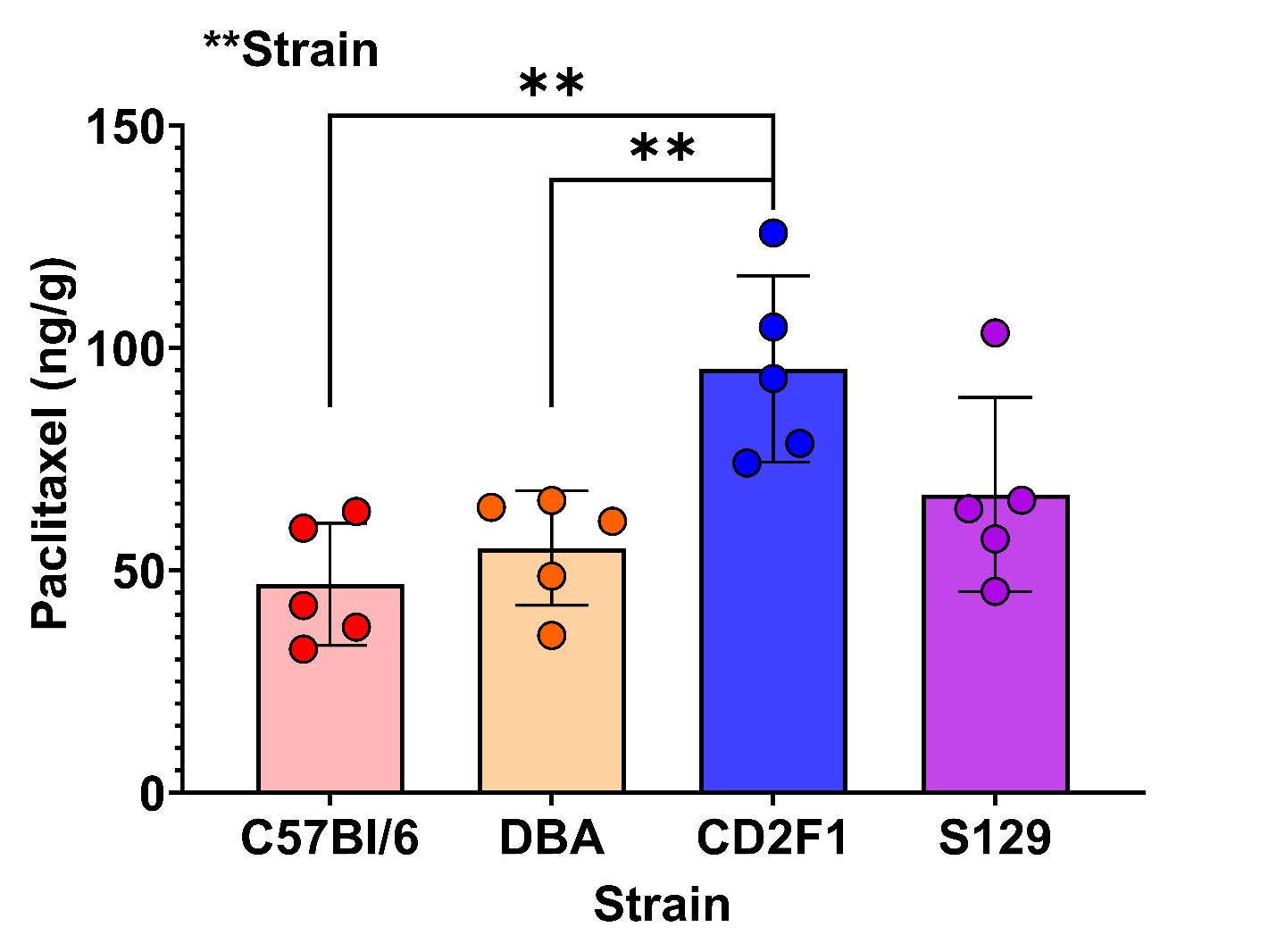


**Supplemental Figure 1**: Pilot experiment comparing concentrations of paclitaxel within murine DRG after a single 10 mg/kg intravenous bolus, 24 h after administration. (n=5 per group, error bars represent SD). **P<0.01


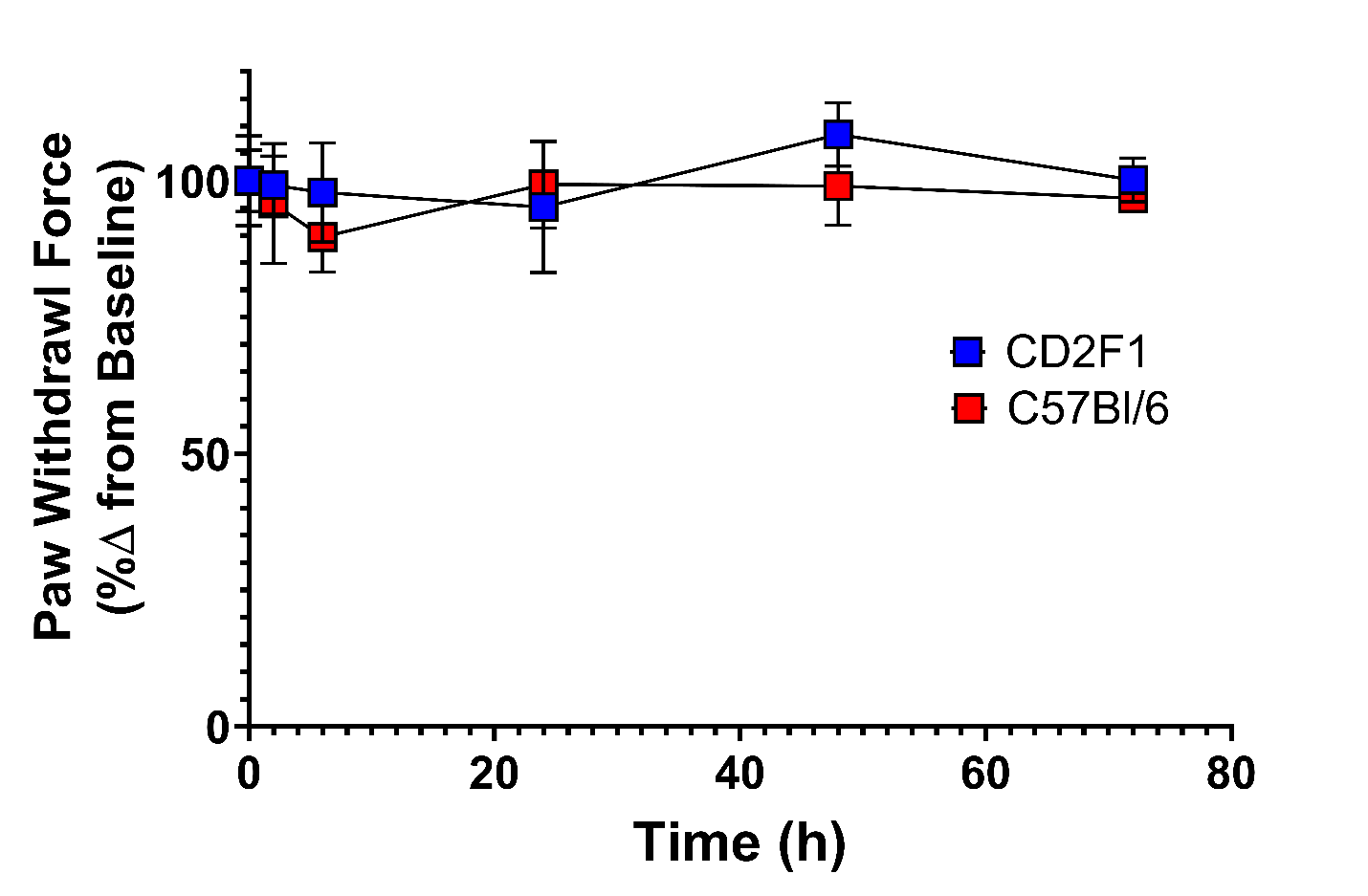


**Supplemental Figure 2:** VFH in CD2F1 and C57Bl/6 mice following a single intravenous bolus of vehicle, expressed as a percent change from baseline (n=6 per group, error bars represent SD).

**
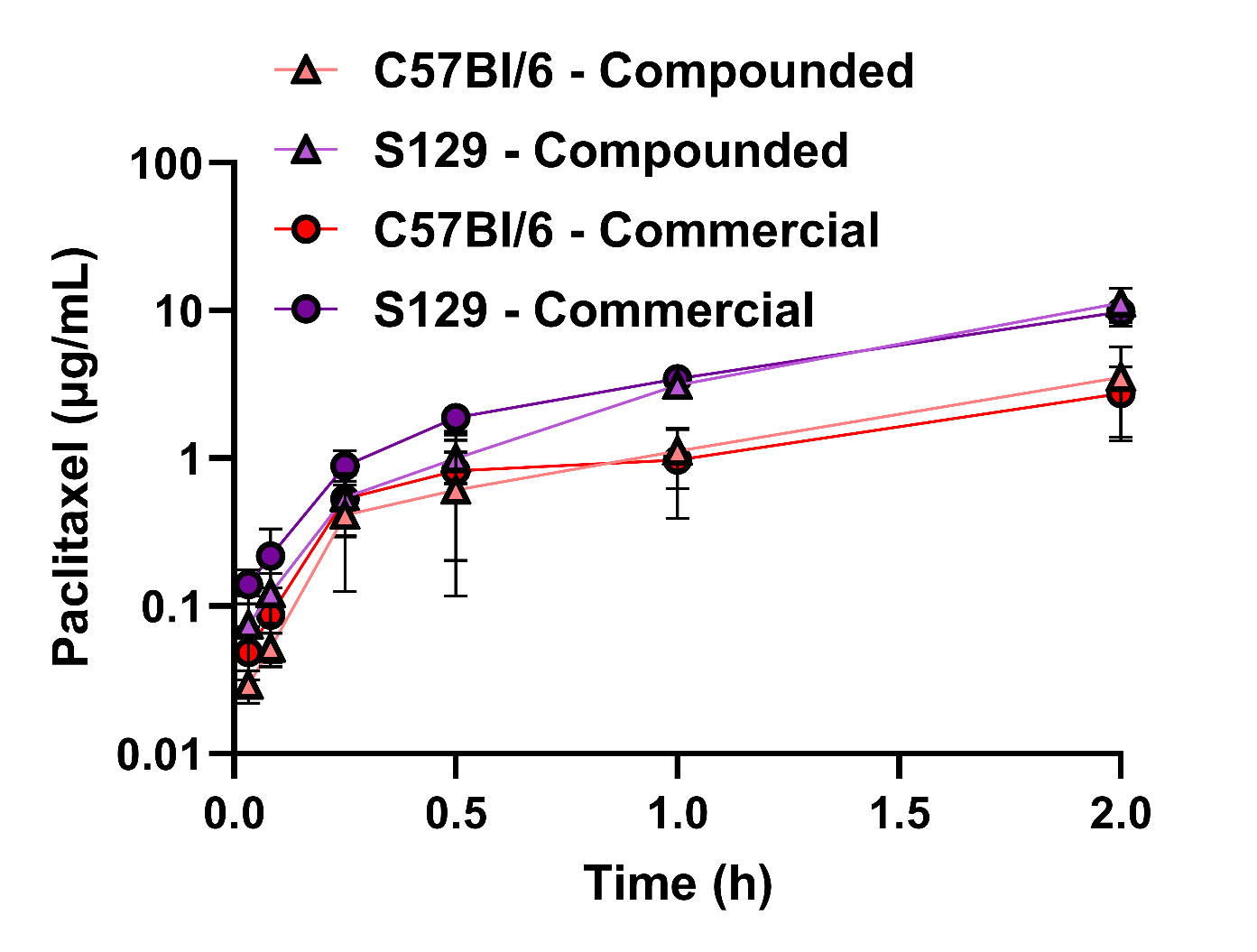
**

**Supplemental Figure 3**: Concentration-time curve of paclitaxel formulated commercially or compounded using powder in-house after a single 10 mg/kg IP dose. (n=5 per group, error bars represent SD).


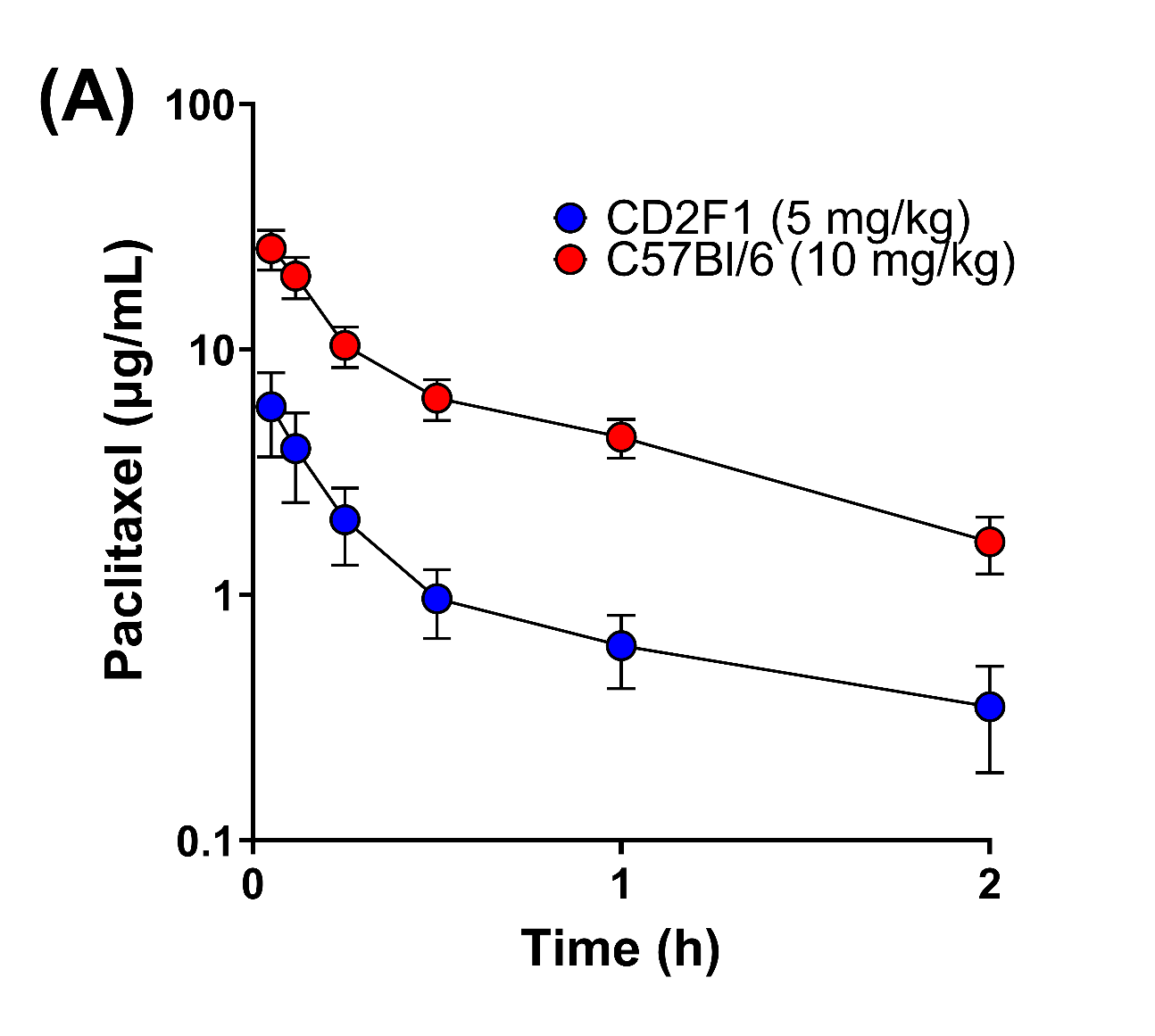


**Supplemental Figure 4:** **Dose modulation of paclitaxel.** **(A)** Plasma concentration–time profiles of paclitaxel administered intravenously to CD2F1 (5 mg/kg) and C57Bl/6 mice (10 mg/kg).

| Strain | Sex | Route | n | Formulation | Dose (mg/kg) | C_max_ (µg/ml) | AUC_(0-2h)_ (µg*hr/ml) |
| --- | --- | --- | --- | --- | --- | --- | --- |
| C57Bl/6 | F | IP | 5 | Compounded | 10 | 3.54 ± 2.41 | 2.86 ± 1.54 |
| C57Bl/6 | F | IP | 5 | Commercial | 10 | 2.73 ± 1.41 | 2.46 ± 1.14 |
| S129 | F | IP | 5 | Compounded | 10 | 11.2 ± 2.93 | 8.52 ± 1.47 |
| S129 | F | IP | 5 | Commercial | 10 | 9.8 ± 1.95 | 8.42 ± 1.43 |

**Supplemental Table 1**: Pharmacokinetic parameters for commercial and in-house compounded formulations of paclitaxel.

| Strain | Sex | Route | n | Dose (mg/kg) | C_max_ (µg/ml) | AUC_(0-2h)_ (µg*hr/ml) |
| --- | --- | --- | --- | --- | --- | --- |
| C57Bl/6 | F | IV | 5 | 10 | 26.0 ± 10.51 | 11.0 ± 4.47 |
| CD2F1 | F | IV | 4 | 5 | 5.8 ± 4.92 | 2.0 ± 1.57 |

**Supplemental Table 2**: Pharmacokinetic parameters for plasma exposure following a 5 or 10 mg/kg dose of paclitaxel.
